# Mucinous Borderline Tumor Associated with Mesonephric-like Proliferation: Further Evidence for a Possible New Origin of Ovarian Mucinous Neoplasms

**DOI:** 10.3390/diagnostics12081901

**Published:** 2022-08-05

**Authors:** Jie Zhang, Yuling Dong, Xiaoqiu Zhou, Brian S. Finkelman, Deyin Xing

**Affiliations:** 1 Department of Pathology, Maternal and Child Health Care Hospital of Shandong Province, Jinan 250014, China; 2 Department of Pathology, The Johns Hopkins Medical Institutions, Baltimore, MD 21231, USA; 3 Department of Oncology, The Johns Hopkins Medical Institutions, Baltimore, MD 21231, USA; 4 Department of Gynecology and Obstetrics, The Johns Hopkins Medical Institutions, Baltimore, MD 21231, USA

**Keywords:** mesonephric-like proliferation, mucinous borderline tumor, mucinous metaplasia, ovary

## Abstract

Some ovarian mucinous tumors are thought to originate from Brenner tumors and teratomas; however, data are limited on what could be the origin for the remaining tumors. We report a new case of ovarian mucinous borderline tumor/atypical proliferative mucinous tumor (MBT/APMT) co-existing with a mesonephric-like proliferation (MLP)/mesonephric-like hyperplasia (MLH). The patient was a 58-year-old woman who presented with a pelvic mass and abdominal pain. Pathology demonstrated an 11 cm MBT/APMT in the left ovary. In addition, the tumor contained one focal area (<1% of total tumor volume) of MLP/hyperplasia adjacent to, or intimately admixed with, mucinous epithelium, with an immunophenotype of diffuse Pax8 and Gata3 expression and negative TTF-1, ER, and PR staining. Pax8 was also weakly positive in the MBT/APMT component. Some mesonephric-like glands partially exhibited gastrointestinal-type mucinous metaplasia/differentiation. A polymerase chain reaction (PCR)-based Sanger sequencing demonstrated that a *KRAS* G12V mutation was present in both MLP/MLH and MBT/APMT components, providing further evidence to support their clonal relationship. We previously reported a series of similar cases and demonstrated a novel association between MLP, mesonephric-like adenocarcinoma and ovarian mucinous tumor. It is conceivable that benign MLPs may have ability to differentiate to lineage-specific mucinous lesions, and, as such, they may serve as a possible new origin of some ovarian mucinous neoplasms; in particular, Pax8-positive tumors. The current case provides additional evidence to support this theory.

## 1. Introduction

Primary ovarian mucinous tumors are thought to progress stepwise from cystadenomas, to mucinous borderline tumors/atypical proliferative mucinous tumors (MBT/APMT), and to intraepithelial and/or invasive carcinomas, representing a biological continuum in the sequence of carcinogenesis [1,2,3]. Although studies have shown that most epithelial ovarian tumors develop from Müllerian-derived precursors, ovarian mucinous tumors typically lack a Müllerian phenotype [4,5,6,7]. It has been postulated that a portion of mucinous tumors are derived from Brenner tumors or teratomas and molecular studies have indicated a clonal relationship to support this hypothesis [6,8,9,10,11,12]. Brenner tumors are composed of nests of transitional-type epithelium (resembling urothelial cells) within a fibromatous stroma. These nests frequently contain central cavities lined by mucinous epithelium that, if overgrown, could result in the formation of a mucinous tumor. Teratomas are thought to arise from the primordial germ cell and are composed of mature or immature tissues derived from one, two or three germ layers. In theory, they can give rise to any type of neoplasm, including mucinous tumors, since all three germ layers have the potential to undergo lineage-specific tumorigenesis. It has been reported that ovarian mucinous tumors of either origin exhibit a comparable gastrointestinal immunophenotype during progression and show early mutations in *KRAS* and *TP53* [8].

As a newly described entity which was recently added to the 2020 World Health Organization (WHO) Classification of Female Genital Tumors [3,13], mesonephric-like adenocarcinoma (MLA) in the endometrium and ovary displays similar morphology, immuno-profile, and genetic alterations, to mesonephric adenocarcinoma of the uterine cervix. While the latter is thought to arise from mesonephric remnants, the former lacks evident association with these Wolffian-type precursor lesions. Rather, some MLAs in the ovary are related to Müllerian-type lesions, in particular, serous borderline tumor and endometriosis, providing evidence to support the notion that MLAs may be derived from transdifferentiation of Müllerian tissue into a Wolffian/mesonephric lineage [14,15,16,17,18]. Typically, mesonephric adenocarcinoma and MLAs lack mucinous and squamous features/differentiation [19].

We previously reported a series of ovarian mucinous tumors (1 mucinous cystadenofibroma and 3 MBTs/APMTs) co-existing with mesonephric-like lesions, all of which contained mesonephric-like proliferation (MLP)/mesonephric-like hyperplasia (MLH) which focally displayed gastrointestinal-type mucinous metaplasia/differentiation [20]. Speculatively, benign MLPs may have ability to differentiate to lineage-specific mucinous lesions, and, as such, they may serve as a possible new origin of some ovarian mucinous neoplasms. Here, we report a new case of ovarian MBT/APMT associated with MLP/MLH, which provides further evidence to support this theory.

## 2. Materials and Methods

### 2.1. Patient Information

The patient was a 58-year-old woman who presented with a pelvic mass and abdominal pain without significant past medical history. An ultrasound examination revealed a 13.2 cm × 12.8 cm × 11.0 cm multiloculated cystic mass with thin and thick septations and solid components arising from the left adnexa. Only minimal vascularity was noted in the septations or solid areas. A total abdominal hysterectomy and bilateral salpingo oophorectomy, omentectomy, appendectomy, and staging biopsies were subsequently performed. The patient had no evidence of recurrent/residual disease by imaging at 5 months follow-up.

### 2.2. Immunohistochemistry

Immunohistochemical staining was performed on formalin-fixed, paraffin-embedded tissue sections in the Immunopathology Laboratory in the Department of Pathology, Maternal and Child Health Care Hospital of Shandong Province, Shandong, China. Prediluted antibodies from ZSGB-BIO, Beijing, China, included: AR (EP120), CDX2 (EP25), CK20 (EP23), Gata3 (EP368), p53 (DO-7), Pax8 (OTI6H8), TTF-1 (SPT24), Villin (UMAB230); Prediluted antibodies from MXB Biotechnologies, Fuzhou, China, included: CD10 (MX002), CK7 (OV-TL12/30), ER (SP1), PR (SP2).

### 2.3. DNA Extraction and PCR-Based Sanger Sequencing

Formalin-fixed paraffin-embedded (FFPE) tumor and corresponding normal tissues were identified by H&E staining and were subsequently macro-dissected (with tumor elements accounting for about 60% or more of the section area), and genomic DNA was extracted using a QIAamp DNA FFPE Tissue Kit with an adopted protocol (Qiagen, Valencia, CA, USA). Hotspot mutation in the *KRAS* codon 12 was assessed by polymerase chain reaction (PCR)-based Sanger sequencing [21]. Briefly, 50 ng of DNA was amplified by PCR with *Taq* DNA Polymerase and Standard *Taq* Buffer (New England BioLabs, Ipswich, MA, USA). The following primers were used for amplification: 2F1: 5′-GTGTATTAACCTTATGTGTGACA-3′, 2R1: 5′-TGGTCAGAGAAACCTTTATCTG-3′, and 2R2: 5′-TGGTCCTGCACCAGTAATATGC-3′. The PCR products amplified by 2F1/2R1 primers were diluted 10 times and were used as templates for the second PCR amplification (nested PCR) utilizing another pair of primers 2F1/2R2. DNA sequencing of the purified DNA products was performed using the ABI 3730 High-Throughput DNA Sequencer. 

## 3. Results

**Figure 1:**
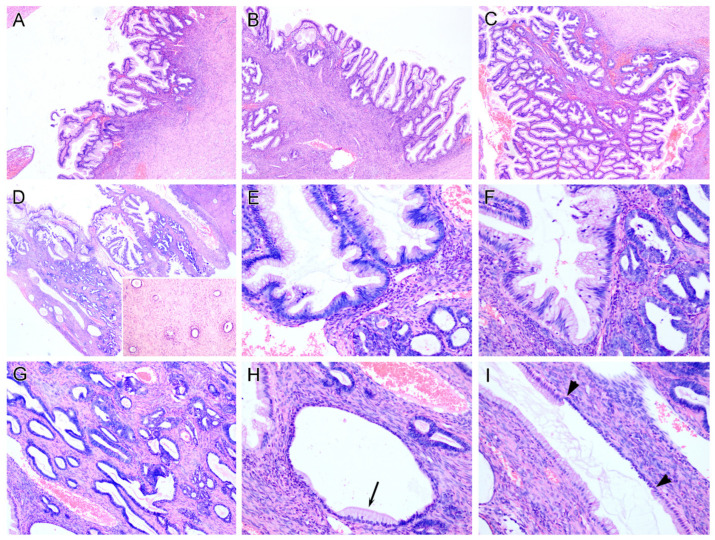
Ovarian mucinous borderline tumor/atypical proliferative mucinous tumor (MBT/APMT) associated with mesonephric-like hyperplasia. The vast majority of the ovarian tumor was composed of MBT/APMT (**A**–**C**). In one focal area (less than 1% of the total tumor volume), clusters of an atypical glandular proliferation were found adjacent to, or intimately admixed with, the mucinous glands (**D**–**F**). Some of the proliferative tubules displayed glandular enlargement, crowding, irregular shape and pseudo-stratification, indicating a hyperplastic process (**G**). On high power, some mesonephric-like glands partially exhibited gastrointestinal-type mucinous metaplasia/differentiation (**H**), arrow indicates metaplastic mucinous epithelium; (**I**), arrow head indicates the junction between cuboidal mesonephric epithelium and columnar mucinous epithelium). In some areas away from the focus of hyperplasia, individual benign mesonephric/mesonephric-like glands, lacking hyperplastic features, were present, which were not associated with the mucinous-type epithelium ((**D**), inset).

The gross examination of the left ovary revealed an 11.0 cm × 8.5 cm × 2.0 cm collapsed cystic lesion with the cyst wall thickness ranging from 0.3 to 0.4 cm. A tan-yellow, thickened area measuring 1.0 cm in greatest dimension was noted in the cyst wall, representing normal ovarian tissue. The lesion was confined in the ovary without extraovarian involvement. Microscopic examination revealed that the vast majority of the ovarian tumor was composed of MBT/APMT characterized by gastrointestinal-type epithelium ranging from a single layer of mucinous cells to varying degrees of stratification, tufting, and villous or finger-like projections (Figure 1A–C). The tumor cells contained abundant gastrointestinal-type mucin and displayed mild to moderate nuclear enlargement and hyperchromasia, but overt evidence of intraepithelial carcinoma was not seen. 

Of 17 submitted blocks (some blocks contained 2–3 pieces of tumor tissue) from the ovarian tumor, one focal area (less than 1% of total tumor volume) displayed clusters of an atypical glandular proliferation adjacent to, or intimately admixed with, the mucinous glands (Figure 1D–F). This glandular proliferation consisted of small, round tubules with luminal eosinophilic secretions but without a lobular arrangement (Figure 1E). The tubules were lined by non-stratified cuboidal cells with minimal nuclear atypia and lacking evident mitosis. No stromal reaction was seen. Although not surrounding a central duct, these tubules cytologically and architecturally resembled mesonephric remnants of the uterine cervix, and were best designated as “MLP/MLH”. Some of the proliferative tubules displayed features of hyperplasia, characterized by glandular enlargement, crowding, irregular shapes and pseudo-stratification, but lacking definitive features of adenocarcinoma (Figure 1G). On high power, some mesonephric-like glands showed focal gastrointestinal-type mucinous metaplasia/differentiation (Figure 1H,I). In some areas away from the focus of MLH, a few individual benign mesonephric/mesonephric-like glands, lacking hyperplastic features, were present, which were not associated with the mucinous-type epithelium (Figure 1D, inset).

**Figure 2:**
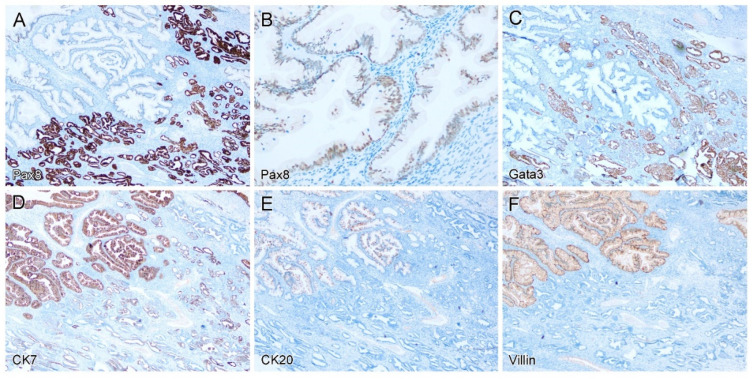
Immunohistochemical findings. The mesonephric-like proliferations/hyperplasia were diffusely positive for Pax8 (**A**), Gata3 (**C**), and focally positive for CK7 (**D**), but negative for CK20 (**E**) and villin (**F**). By contrast, the mucinous epithelium was weakly positive for Pax8 (**B**), diffusely positive for CK7 (**D**) and villin (**F**), focally positive for CK20 (**E**), but negative for Gata3 (**C**).

Immunohistochemically, the MLP/MLH was diffusely positive for Pax8 (Figure 2A), Gata3 (Figure 2C), and focally positive for CK7 (Figure 2D), but negative for CK20 (Figure 2E), villin (Figure 2F) and CDX2 (not shown). The mucinous epithelium was also weakly positive for Pax8 (Figure 2B), diffusely positive for CK7 (Figure 2D) and villin (Figure 2F), and focally positive for CK20 (Figure 2E) and CDX2 (not shown), but negative for Gata3 (Figure 2C). Both components were negative for ER, PR, AR, TTF-1 and CD10. Immunostaining for p53 showed a wild-type pattern throughout.

**Figure 3:**
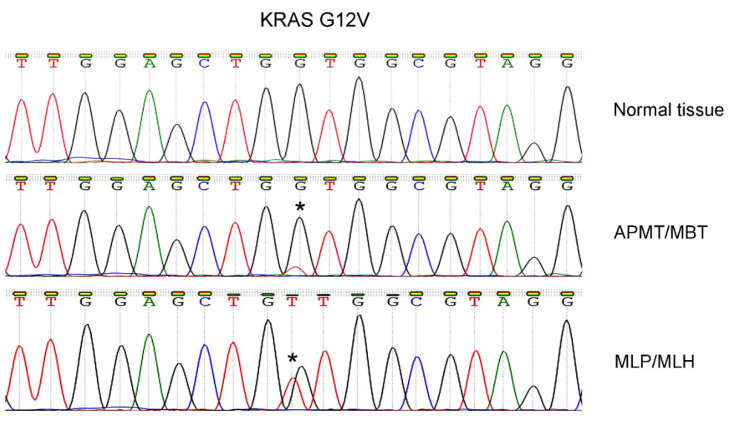
Molecular study/Sanger sequencing showed that the *KRAS* G12V mutation was present in both atypical proliferative mucinous tumor/mucinous borderline tumor/(APMT/MBT) and mesonephric-like proliferation/mesonephric-like hyperplasia (MLP/MLH), indicating their clonal origin. Star (*) indicates mutational site.

To assess *KRAS* codon 12 hotspot mutation, polymerase chain reaction (PCR)-based Sanger sequencing was performed [20,21]. DNA sequencing of the purified DNA products showed that the *KRAS* G12V mutation was present in both MLP/MLH and MBT/APMT components, indicating a clonal origin (Figure 3). 

## 4. Review of Published Cases of Ovarian Combined MBT/APMT and MLP/MLA

**Figure 4:**
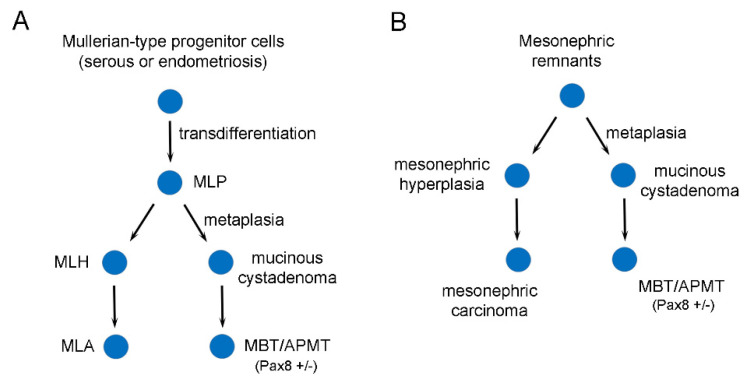
Histopathogenic models for mixed mesonephric and mucinous tumors in the ovary. (**A**) A model of Müllerian/mesonephric-like origin. (**B**) A model of Wolffian/mesonephric origin. MBT/APMT: mucinous borderline tumor/atypical proliferative mucinous tumor; MLA: mesonephric-like adenocarcinoma; MLH: mesonephric-like hyperplasia; MLP: mesonephric-like proliferation.

Including the current case, a total of seven ovarian combined mucinous tumors and mesonephric-like lesions have been reported [14,20]. Clinicopathological features and selected immunohistochemical findings are summarized in Table 1. The ovarian mucinous tumors included 6 MBT/APMTs and 1 mucinous cystadenofibroma; the coexisting mesonephric-like lesions included 4 mesonephric-like adenocarcinomas (MLAs), 2 MLHs and 1 pure benign MLP. The patients ranged in age from 52 to 67 years (mean, 58; median, 58). Tumors were all unilateral (left ovary, 4 cases; right ovary, 3 cases), and ranged from 10 to 28 cm (mean, 15; median, 12) in greatest dimension. Similar to the models for ovarian combined serous tumors and mesonephric-like lesions [21], we also propose two models to explain the histopathogenesis of mixed mucinous tumors (Figure 4).

## 5. Discussion

To our knowledge, this case represents the seventh published case of ovarian mucinous tumors coexisting with mesonephric-like lesions [14,20]. The median age of the patents with these mixed tumors was 58 years, which is similar to those reported in mesonephric carcinoma of the uterine cervix and pure MLAs of endometrium and ovary, but about one decade older on average than those of pure MBT/APMT [2,3]. A similar finding has been reported in mixed ovarian serous tumors and mesonephric-like lesions [14,16,17,18,21].

In this case, the vast majority of the tumor was typical MBT/APMT lined by gastrointestinal-type epithelium. In contrast, the small area of non-MBT/APMT atypical glandular proliferation displayed typical mesonephric morphology with Pax8 and Gata3 expression. The degree of glandular crowding was consistent with hyperplasia but considered insufficient to establish a diagnosis of MLA. Interestingly, a benign MLP was identified in all cases in our previous study [20] as well as in the current case, and all MLPs displayed focal gastrointestinal-type mucinous metaplasia/differentiation and were intimately associated with the mucinous glands. We speculate that the mesonephric epithelium is able to, through metaplasia, transdifferentiate to the mucinous epithelium, which then can give rise to a mucinous tumor. As such, we propose that MLPs are a possible new origin of some ovarian mucinous tumors.

In the current literature, it has been well accepted that Brenner tumors and teratomas are possible sources of ovarian mucinous tumors [1,2]. If teratomatous in origin, the tumor is usually low-grade and displays features similar to that of a low-grade mucinous neoplasm of appendiceal origin with a typical CK7−/CK20+ coordinate expression profile [9]. This immunostaining pattern differs from the more common CK7+/CK20− variable immuno-profile of primary ovarian mucinous tumors of surface epithelial type [12,22,23]. It has been speculated that the majority of the latter arise in Brenner tumors as an overgrowth of the mucinous component, which often grows to form the dominant mass [1,6,24]. We suspect that the histopathogenic pattern of mucinous tumors of mesonephric origin is similar to those of Brenner tumor origin since both often display mucinous metaplasia. Immunohistochemically, all mucinous tumors in our series showed a diffuse CK7 and focal or negative CK20 staining pattern, similar to mucinous tumors derived from Brenner tumors.

The histopathogenesis of Brenner tumors is thought to be related to Walthard rests which are nests of transitional-type epithelium that have invaginated into underlying para-tubal tissue [24,25,26]. The evidence supporting the link between Brenner tumor and Walthard rests includes similar morphology and a shared immuno-profile, illustrated by diffuse expression of Gata3 and lack of Pax8 and Pax2 expression [27]. It has been reported that Pax8, a Müllerian marker, is present in up to half of ovarian mucinous tumors [28]; however, this marker is rarely expressed in those with a Brenner tumor origin [6]. If these Pax8-positive mucinous tumors are indeed of Brenner tumor origin, it is difficult to explain how a Pax8-negative precursor lesion would subsequently gain expression of Pax8 in more terminally differentiated mucinous cells. 

By contrast, the observation in our case provides a plausible explanation for a subset of Pax8-positive ovarian mucinous tumors, because if these tumors arise from mesonephric remnants/proliferations that commonly show diffuse expression for Pax8 and Gata3, some of them may still retain a variable degree of Pax8 expression, whereas nearly all cases lose Gata3 expression. In the published cases [14,20] and our current case, 5 (71%) of 7 mucinous tumors showed focal Pax8 expression (Table 1). Interestingly, in one previously reported case (OV75), the mucinous component was positive for Gata3 and TTF-1 with an intensity weaker than what was found in the mesonephric-like component, indicating an ongoing transitional process [14]. In our case, MLP/MLH was negative for TTF-1 and CD10, which does not refute interpretation as a mesonephric/mesonephric-like lesion. In fact, it has been reported that Gata3 and TTF-1 usually exhibit a reverse staining pattern in mesonephric carcinoma [29]. Likewise, a varied degree of CD10 staining has been described in these lesions. For example, a recent study showed that 7 (47%) of 15 tested mesonephric adenocarcinomas and MLAs lacked CD10 expression [14].

One may argue that, if MLPs were indeed an origin of some mucinous tumors, it might be limited to very rare instances, since the evident link between these two lesions has not been well reported. However, we suspect that, similar to mucinous tumors of either teratomatous or Brenner tumor origin, pure mucinous tumors could arise from mesonephric-type precursor lesions that are then compressed and obliterated by the expanding mucinous tumor [6]. This scenario could make it more difficult to identify the associated mesonephric proliferation in histologic sections of mucinous tumors.

Despite the difference in the morphology and immunophenotype, clonal relationship between morphologically distinct mucinous tumor and MLA has been confirmed at a molecular level [14,20]. Likewise, our current case revealed a *KRAS* G12V mutation in both the MBT/APMT and MLP/MLH components, providing further evidence to support their clonal relationship. We have proposed a histopathogenic model for this type of mixed tumor and we further discuss this theory in the current case study. It has been reported that mesonephric-like differentiation/hyperplasia, not MLAs, can be associated with ovarian serous or endometrioid borderline tumors [21]. In theory, a Müllerian-type progenitor cell in a serous tumor or endometriosis is able to differentiate into mesonephric lineage through trans-differentiation (Figure 4A). The latter may undergo gastrointestinal-type mucinous metaplasia, subsequently giving rise to mucinous cystadenoma/cystadenofibroma and MBT/APMT. Some MLPs stay in the same lineage, acquire additional genetic alterations, and produce MLH and MLA. On the other hand, Müllerian-type lesion is not identified in any of these cases. Therefore, the presence of true mesonephric remnants in these locations is possible (Figure 4B). If mesonephric remnants indeed exist in the ovary and/or para-ovarian tissue, then an adenocarcinoma arising in this lineage may represent a true “mesonephric carcinoma”, rather than an MLA.

## 6. Conclusions

We report a new case of MBT/APMT with co-existent mesonephric lesion/hyperplasia in the ovary. Similar to the cases we reported previously, the current case contained benign MLPs which partially showed gastrointestinal-type mucinous metaplasia/differentiation. We postulate that MLPs are a possible new origin of some ovarian mucinous tumors, particularly, Pax-8 positive tumors. This case provides additional evidence to support this theory.

## Figures and Tables

**Table 1:** Clinicopathological features and selected immunohistochemical findings.

Case	Age (years)	Tumor Site	Tumor Size (cm)	Mucinous Component	Mesonephric Component	Mucinous Component (Pax8)	Mesonephric Component (Pax8)	Mucinous Component (Gata3)	Mesonephric Component (Gata3)
1 Nilforoushan et al. [20]	56	Left ovary	12	MBT/APMT	MLH in a background of MLP	Negative	Positive	Negative	Positive
2 Nilforoushan et al. [20]	67	Right ovary	28	MBT/APMT	Microscopic foci of MLP	Focally positive	Positive	Negative	Positive
3 Nilforoushan et al. [20]	58	Left ovary	12	Mucinous cystadenofibroma	MLA in a background of MLP	Focally positive	Positive	Negative	Positive
4 Nilforoushan et al. [20]	55	Left ovary	13	MBT/APMT	MLA in a background of MLP	Negative	Positive	Negative	Positive
5 da Silva et al. [14]	52	Right ovary	18.5	MBT/APMT	MLA	Positive	Positive	Negative	Positive
6 da Silva et al. [14]	60	Right ovary	10	MBT/APMT	MLA	Weakly positive	Positive	Weakly positive	Positive
7 This study	58	Left ovary	11	MBT/APMT	MLH in a background of MLP	Weakly positive	Positive	Negative	Positive

APMT: atypical proliferative mucinous tumor; MBT: mucinous borderline tumor; MLA: mesonephric-like adenocarcinoma; MLH: mesonephric-like hyperplasia; MLP: mesonephric-like proliferation.
